# Areas of Potential Impact of the Patient Protection and Affordable Care Act on EMS: A Synthesis of the Literature

**DOI:** 10.5811/westjem.2017.1.32997

**Published:** 2017-03-13

**Authors:** Daniel G. Ostermayer, Charles A. Brown, William G. Fernandez, Emily Couvillon

**Affiliations:** * The University of Texas Health Science Center Houston, Department of Emergency Medicine, Houston, Texas; † Houston Fire Department, Houston, Texas; ‡ McGovern Medical School, Houston, Texas; § Texas Medical Center Library, Houston, Texas

## Abstract

**Introduction:**

This comprehensive review synthesizes the existing literature on the Patient Protection and Affordable Care Act (ACA) as it relates to emergency medical services (EMS) in order to provide guidance for navigating current and future healthcare changes.

**Methods:**

We conducted a comprehensive review to identify all existing literature related to the ACA and EMS and all sections within the federal law pertaining to EMS.

**Results:**

Many changes enacted by the ACA directly affect emergency care with potential indirect effects on EMS systems. New Medicaid enrollees and changes to existing coverage plans may alter EMS transport volumes. Reimbursement changes such as adjustments to the ambulance inflation factor (AIF) alter the yearly increases in EMS reimbursement by incorporating the multifactor productivity value into yearly reimbursement adjustments. New initiatives, funded by the Center for Medicare & Medicaid Innovation are exploring novel and cost-effective prehospital care delivery opportunities while EMS agencies individually explore partnerships with healthcare systems.

**Conclusion:**

EMS systems should be aware of the direct and indirect impact of ACA on prehospital care due to the potential for changes in financial reimbursement, acuity and volume changes, and ongoing new care delivery initiatives.

## INTRODUCTION

### Background and ACA History

In the United States, the Patient Protection and Affordable Care Act (ACA), signed into law on March 23, 2010, primarily aimed to expand health insurance coverage, improve quality reporting, and reduce overall costs by encouraging primary care.^1^ The ACA is expected to improve access to healthcare by increasing health insurance enrollment by an estimated 30 million people by the year 2021 via both mandates and subsidies.^2^–^4^ In 2011, emergency medical services (EMS) transported over 21 million people to emergency departments (ED). Of the 136.3 million ED visits, 15.7% arrived by ambulance.^5^

Areas of change in emergency medicine identified by a prior review included a greater proportion of Medicaid-insured patients, changes in patient volume, and variable increases in acuity.^6^ Although these changes are directly studied in relation to patients presenting to the ED, EMS agencies have already begun to implement and propose adaptations that respond to these observed changes.^7^–^9^ This comprehensive review synthesizes the existing literature regarding ACA-related changes in emergency care that impact EMS systems and specific measures within the ACA that have the potential to directly impact EMS systems.

## MATERIALS AND METHODS

This comprehensive review was limited to the English language due to the nature of the subject matter. We searched databases PubMed, EMBASE, Ovid Healthstar, CINAHL and the Cochrane Library for articles published between January 2006 and March 2016 using the following search filter: (“Emergency Medical Services” OR “prehospital” OR “Hospital Emergency Service” OR “Medical Device Legislation” OR “Emergency Medical Service Communication Systems” OR “Emergency Medical Technicians” OR “paramedic” OR “paramedics” OR “paramedicine” OR “Ambulances” OR “ER” OR “EMS” OR “ED” OR “EMT” OR “helicopter” OR “HEMS”) AND (“Patient Protection and Affordable Care Act” OR “affordable care act” OR “ACA”). We identified 435 publications on the subject matter after adding 30 additional citations discovered through a grey literature search.

The citations were combined in Refworks and reviewed manually, resulting in the exclusion of 90 duplicate articles. Of the remaining 345 articles, we excluded 259 after screening the titles and abstracts due to irrelevance to emergency care or EMS. The full text of all the remaining 86 articles was reviewed independently by three authors (DO, CB, and BF) and scored as eligible or ineligible for inclusion. Articles without unanimous approval were determined for inclusion by majority non-anonymous, in-person voting. We excluded six articles based on lack of applicability to emergency care and located six additional articles by reviewing the bibliographies of the included articles. In addition, the sections and provisions of the final publication of the ACA (Public Law 111-148) that directly apply to emergency services were identified via a free-text match using the same literature search terms.

## RESULTS

**Figure 1:**
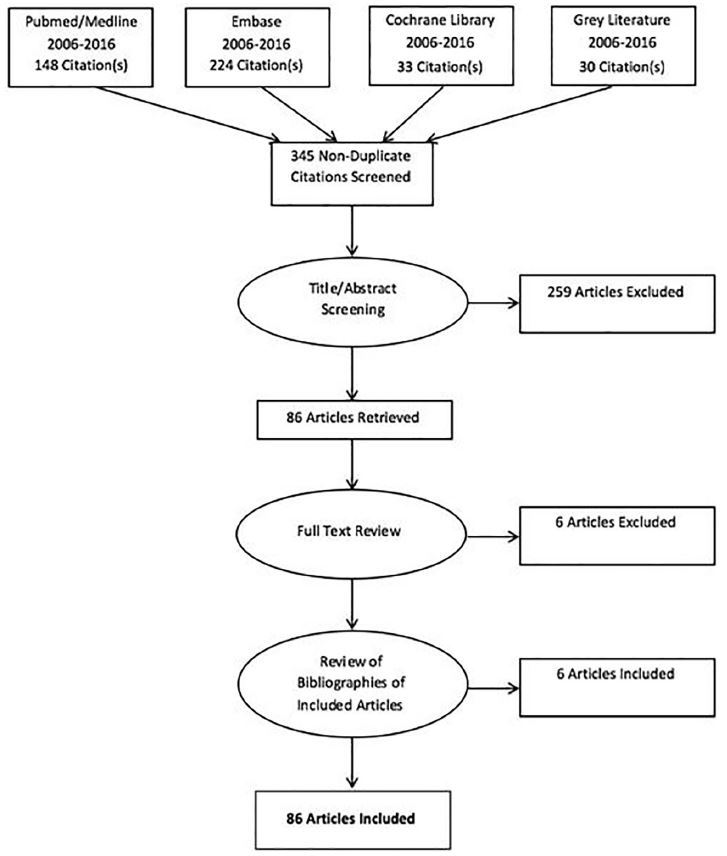
PRISMA flowchart of systematic literature review of changes to emergency care related to the Affordable Care Act that directly affect emergency medical services.

The final review included 86 publications (Figure 1). The review of the ACA identified the following sections that directly mention emergency care and EMS services (Table 1).

## DISCUSSION

### Patient Usage and Access to Care

A major goal of the ACA was to reduce the use of ED care for non-urgent conditions and promote primary care utilization.^10^ Prior to ACA implementation, 15.4 – 16.3%^11^–^17^ of ED patients arrived by ambulance. Data from states that have implemented Medicaid expansion and from those that had implemented similar health insurance reform programs prior to the ACA suggest that ED volume continues to increase despite expanded insurance.^18^–^20^ The direct effects of healthcare expansion efforts on EMS usage have not been described, Due to lack of EMS-specific acuity and volume data, we used ED data as a surrogate for EMS acuity and volume when reviewing publications.

### Medicaid Enrollees Acuity and Volume

Local changes in patient acuity may depend upon the proportions of new Medicaid recipients. New recipients of Medicaid may have a greater need for healthcare after previously deferring care due to lack of insurance.^19^ Data from Oregon and Wisconsin, where Medicaid was expanded to a specific group of the population, demonstrated an increase in ED use of 40% and 46%, respectively.^21^,^22^ The initial transient increase in ED utilization was shown to level off after 18 months during implementation of a program in California that expanded Medicaid early to future potential enrollees.^23^ In areas with a similar Medicaid population, EMS transport volume as a percentage of overall ED volume may increase in the near future and may experience potentially larger than normal short-term increases followed by a gradual long-term taper.

**Population Health Research Capsule**

What do we already know about this issue?

Previously identified effects of the Affordable Care Act on emergency department care include a greater proportion of Medicaid-insured patients, changes in patient volume, and variable increases in acuity.

What was the research question?

What specific areas of emergency medical services are potentially impacted by the ACA?

What was the major finding of the study?

EMS may experience changes in volume and in reimbursement due to a new payer mix and revisions to the Ambulance Fee Schedule.

How does this improve population health?

As the health insurance landscape of the United States continues to evolve, these provisions within the ACA provide areas for future research and operational focus within EMS systems.

A 10% increase in patient acuity, which was measured by resource needs and clinical complexity, and up to 13.2% increase in number of diagnoses have been noted during the first two quarters after newly enrolled Medicaid recipients gain access to care.^18^ Early Medicaid expansion in California brought an increase in hospital admissions, with the most notable increase coming from those who did not use healthcare resources during the year prior.^23^ Similar to volume, local acuity changes may change proportional to the quantity of new Medicaid enrollees who were previously underinsured.

### Deductible effects on usage

Up to 85% of the plans chosen in health exchanges now contain an increased deductible,^24^ which may incentivize individuals to defer seeking care until an absolute emergency. Among individuals with lower socioeconomic status (SES) who purchased low premium, high-deductible plans, high-acuity ED visits decreased 24.5% in the first year after enrollment and decreased another 7.4% in the second year. Similarly, hospitalizations among those with lower SES dropped by 23% in the first year but increased the need for subsequent hospitalizations. This is in contrast to individuals of high SES with high-deductible plans who had no significant change in ED visits or hospitalizations.^25^ As such, the usage of ambulance services as a proportion of patients seeking emergency care should change based on the proportion of SES individuals in the EMS catchment area who have purchased high-deductible health insurance plans.

## HEALTHCARE QUALITY ASSESSMENTS

### Readmissions

In an attempt to improve the quality of healthcare, the Centers for Medicaid and Medicare Services (CMS) began allocating additional funds to hospitals in 2013 for those meeting a set of quality standards. Hospitals are graded on standards that include health outcomes, patient safety, efficiency, equity, and patient satisfaction. Section 3025 of the ACA established the Hospital Readmissions Reduction Program (HRRP) to penalize reimbursement based on readmissions for a specific set of diagnoses (Table 2). The diagnoses have expanded since 2013 and the percentages of the penalties are also increasing.^26^,^27^ Again, like many other measures, the HRRP applies only to hospitals and does not change EMS reimbursement, but it has offered some new opportunities for EMS to partner with hospital systems in the implementation of readmission reduction programs.

Mobile Integrated Healthcare and Community Paramedicine (MIH-CP) programs are being implemented and evaluated by some EMS systems as a viable option for reducing readmissions and EMS transports.^28^ These programs offer opportunities for EMS to provide healthcare in non-traditional roles using knowledge that is standard among EMS personnel and critical care nursing.^29^ Such models offer unique funding mechanisms such as those demonstrated by MedStar Mobile Healthcare, in which a portion of hospital savings is passed back as reimbursement to an EMS agency if a readmission was prevented within 30 days.^30^

As an example, Medstar performs house visits to educate patients on management of chronic conditions and evaluate for opportunities to decrease unnecessary transports to EDs.^31^ Over a five-year period, this program prevented 1,893 transports to the ED due to 911 calls. The estimates in Medicare savings, however, are small at $21,627.^31^ Another similar program in California at 12 statewide sites uses paramedics working under physician supervision to provide services that include transportation to mental health or urgent care clinics, follow-up care for individuals recently released from the hospital, hospice care, and assistance to frequent EMS utilizers.^32^ Long-term funding and sustainability of these and other similar programs is both uncertain and currently unpublished.

## REIMBURSMENT CHANGES

### Ambulance Fee Schedule

**Figure 2:**
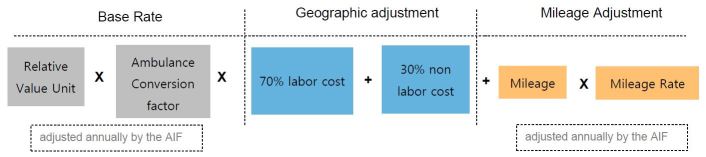
Ambulance fee schedule reimbursement calculation. *AIF,* ambulance inflation factor.

The Centers for Medicare and Medicaid Services (CMS) established a fee schedule for EMS reimbursement in 2002. The established ground-service fee schedule consists of seven levels of services in which a relative value unit (RVU) was established for each level of transport. These RVU values are multiplied by a conversion factor to correlate reimbursement with level of care. There is also an additional mileage fee and adjustment factors that are dependent on the location of service (Figure 2).^33^ The rural bonus, which provides additional reimbursement for rural transport, was extended until 2011 in section 3105 and later extended by Section 104(a) of the Protecting Access to Medicare Act of 2014 to March 31, 2015, and further extended via Section 203 of the Medicare Access and CHIP Reauthorization ACT of 2015 (MACRA) until December 31, 2017. 34,35

Prior to the ACA, the price increases for ambulance payments were equal to a percentage increase in the urban Consumer Price Index (CPI). Going forward, the Ambulance Inflation Factor (AIF) will subtract the nonfarm Multifactor Productivity (MFP) value from the CPI. The nonfarm MFP accounts for economy productivity based on the labor outputs and capital invested. The value incorporates technological innovation and new efficiencies while the CPI simply accounted for price inflation of services. For the first time since the enactment of the ACA, the AIF will be negative. Specifically, the MFP is 0.5 and the CPI is 0.1 so the AIF is adjusted down −0.4 percent.^36^ The overall implication is encouragement to improve productivity along with the remainder of the economy regardless of inflation rates. The main potential issue with the new AIF calculation is that EMS costs are mainly personnel not technological, and the resulting MFP adjustment on an annual basis could negatively impact reimbursement as the productivity of the U.S. economy increases relative to EMS costs and inflation.

### Payer Mix

Based on CMS estimates, the uninsured population in the U.S. is estimated to decrease by 33.8 million people by 2019.^37^ The number of Medicaid patients, however, is estimated to increase, especially in states that have adopted the Medicaid expansion. An analysis including 465 hospitals in 30 different states found a 25% decline in self-pay status.^18^ These changes correlate with the increases in Medicaid and may not occur in non-expansion states.^38^ Under CMS guidelines, which are also frequently followed by private insurance companies, EMS services must transport patients to a hospital to receive reimbursement for their care.^39^ The median cost of EMS transport was $429 in 2010, with median Medicare reimbursement for those transports $464.^40^ It should be expected that these small margins on Medicaid patients will continue and may even shrink based on Ambulance Inflation Factor (AIF) changes. The continued low margins could be offset by the increasing payments from a greater percentage of insured patients, but ultimately depend on the local payer mix.^41^

## NEW INNOVATIONS

### CMMI Awards

Section 3021 of the ACA established the Center of Medicare and Medicaid Innovation (CMMI) to test innovative payment and service-delivery models that aim to reduce expenses and improve costs. A few EMS agencies are currently taking advantage of the grants offered from the CMMI in the form of community paramedicine and mobile integrated healthcare and alternative healthcare destination programs.^42^ Other hospital systems are implementing mobile healthcare without partnering with an EMS agency in order to reduce readmission rates for diseases on the HRRP list. For example, the Icahn School of Medicine at Mount Sinai Mobile Acute Care Team (MACT) received funding to pilot a program using community paramedics, nurses, and physicians to perform home treatment for recently discharged patients in order to reduce 30-day readmission rates.^43^ Such hospital-based MIH-CP programs may also fulfill the community outreach requirements for maintenance of hospital non-profit status.^44^,^45^

### Alternative Destinations

Although EMS is currently reimbursed by Medicare Part B as a transportation service to the nearest healthcare facility, agencies are exploring alternative transport destinations options for 911 calls. Mesa Arizona Fire and Medical Department, a recipient of a CMMI award, is testing a model that involves paramedic and nurse practitioner- or physician assistant-staffed response vehicles. In addition to field treatment and release, the system can divert patients from the ED to alternative transport destinations.^46^ Although controversial, it is estimated through Medicare claims data from 2005 to 2009, that 12.9–16.2% of EMS transports covered by Medicare may have been comprised of patients whose chief complaints could have been treated in a primary care facility. This may have resulted in a $283–$560 million per year savings.^9^,^47^–^50^ The ACA does not provide a means for EMS agencies to receive reimbursement for the emergent transport of patients to a non-emergent care facility. Overall, the financial stability of alternative destination programs remains unknown as most are funded by “add-on” programs or grants until future Medicare and private insurance change reimbursement requirements.^7^,^39^

## OTHER MEASURES

In addition to the previously highlighted changes, multiple sections of the ACA (1204, 1281, 3504, and 5603 and 498D) provide for continued support of EMSC and trauma center funding and research. For the first time in U.S. EMS history, providers are now recognized officially as part of the healthcare workforce via Section 5105. Section 5210 amended Section 203 of the Public Health Service Act (PHSA) to establish the U.S. Public Health Service Ready Reserve Corps (RRC) to provide additional volunteer member availability for response in foreign or domestic public health emergencies. The RRC provides additional resources if needed to assist the regular USPHS Commissioned Corps personnel. The existing PHSA was further amended to establish an Epidemiology and Laboratory Capacity grant program from the Centers for Disease Control and Prevention Division of Vector-borne Diseases. The state- and local government-awarded laboratories will serve to assist public health agencies in the surveillance of infectious disease and biological threats.^1^,^45^

## CURRENT LIMITATIONS AND FURTHER RESEARCH

A major limitation of this review is lack of directly published literature regarding the financial, operational, and clinical effects of the ACA on EMS systems. The majority of published literature relates to ED care and as such was used as a surrogate for predictions related to EMS changes. Future research is needed regarding the long-term effects of healthcare reimbursement and patient insurance changes on EMS systems. Regionalized and national data will allow for more specific conclusions regarding impacts on prehospital care from current healthcare changes and new innovations.

## CONCLUSION

In the wake of the current healthcare reforms initiated in the U.S. by the ACA, potential changes to EMS are largely side effects of inpatient and ED changes. Although EMS and emergency care is directly addressed by the ACA, changes to transport destinations and operations remain unchanged. Modifications to the ambulance fee schedule will impact EMS departments and potentially place negative pressure on revenue. Alternative sources of funding being supported by CMMI grants, such as MIH-CP, may provide future opportunities, although long-term sustainability is uncertain. EMS agencies that partner with hospital systems may benefit from the continued emphasis on patient- and system-centered healthcare quality metrics. Volume and acuity increases will depend upon state Medicaid expansions, local insurance coverage, and socioeconomic demographics.

## Figures and Tables

**Table 1:** Sections in the Patient Protection and Affordable Care Act identified via systematic search that relate to EMS.

Section	Provision	Summary
1281	Grants to states for trauma service availability	Sub-section 4 awards funding for enhanced collaboration between trauma centers and EMS services
1302	Inclusion of emergency services as Essential Health Benefits for exchange-based health plans	Emergency department services are declared core elements of health insurance and insurance coverage is essential
3021	Establishment of the Center for Medicare and Medicaid Innovation (CMMI)	Test innovative payment and service delivery models that decrease cost and improve quality
3024	Independence at home demonstration program	Testing of payment incentives and delivery models for home based care to reduce emergency department visits, improve outcomes, and prevent readmissions and hospitalizations
3101	Increase in physician payment update	Continued yearly update of the ambulance fee schedule
3105	Ambulance Fee Schedule add on payment extension	Extension through January 1, 2011, of the rural bonus for ground ambulance transport
3401	Revision of market-based productivity increases for the ambulance fee schedule	The Consumer Price Index (CPI) is adjusted downward by the Multifactor Productivity score (MFP) to calculate the new Ambulance Inflation Factor (AIF)
3504/1204	Design and Implementation of regionalized systems for emergency care	Grant awards for trauma systems, EMS systems and comprehensive care systems
5603	Reauthorization of the Wakefield Emergency Medical Services for Children Program (EMSC)	Authorized funding of EMSC activities per congressional appropriation
4304	Epidemiology-Laboratory Capacity (ELC) grants from the Centers for Disease Control and Prevention, Division of Vector-borne Diseases	Establishment of grants for surveillance and threat detection for biologic events
498D	Support for emergency medicine research	Support for NIH-funded emergency medicine research
5101	National health care workforce commission	Recognition of the EMS providers as part of the healthcare workforce
5210	Ready Reserve Corps	Establishment of the Ready Reserve Corps for emergency service

*EMS,* emergency medical services; *NIH,* National Institutes of Health.

**Table 2:** Diagnoses tracked for the Hospital Readmission Reduction Program (HRRP).

Year in effect	Diagnosis
2013–2014	Myocardial infarction
	Congestive heart failure
	Pneumonia
2015	Elective hip arthroplasty
	Elective total knee arthroplasty
	Chronic obstructive pulmonary disease
2016	Stroke
2017	Coronary artery bypass graft
